# A Medical Causerie

**Published:** 1916-07-29

**Authors:** 


### A MEDICAL CAUSERIE.

As is the case with most scientific and professional societies, the Medico-Legal Society has not been able to show much activity during the past session. But it has held on its way so far as possible, and has had an excellent president in Professor Harvey Littlejohn, who during the last two years must have made many journeys from Edinburgh to London in order to preside at its meetings. The new president is the Right Honourable Sir Samuel Evans, the general practice of the Society being to elect alternately a lawyer and a doctor to the chair. The new vice-president is Dr. Robert Armstrong-Jones, the well-known lunacy expert. The difficulties of the situation have been illustrated in most of the sections of the Royal Society of Medicine, and not a few have had a very scanty programme. One of the most successful has been the section for the Study of Disease in Children, which, in the person of Dr. J. D. Rolleston, has the good fortune to have a specially capable and courteous honorary secretary. The recent meeting of the Ophthalmological Society, too, was very successful, and both London and the country were well represented. Mr. Jessop was very happy in the presidential chair and, as was to be expected, everyone desired to do him honour.

The attention of surgical experts, and particularly of those whose work consists largely of endeavours to remedy the havoc created by wounds in war, has been strongly attracted by the cinematograph demonstrations before the Royal Society of Medicine of the methods and tools devised for performing bone grafting by Dr. F. H. Albee, of New York. The chief feature of these methods is their astonishing accuracy, rapidity, and ease of execution; and these factors are largely determined by the ingenious adaptation of machinery to tasks which surgeons have hitherto performed mainly by hand-tools. The advances rendered possible by Dr. Albee's inventions can hardly be set bounds to; they must certainly be enormous, and are almost sure to be epoch-making. There is complete unanimity amongst those surgeons who have seen the film-demonstrations as to the enormous value of these machines and methods; and we trust the Army Medical Department will see to it that all its orthopædic experts in military hospitals are supplied with them at the earliest possible moment. Dr. Albee is a surgeon whose work is already well known in British surgical circles, but it is safe to say that nothing he has hitherto accomplished equals his present contribution to surgical science.

The value of the cinematograph for purposes of medical demonstration and teaching is not new and by its aid the procession of various operations have been exhibited in a truly graphic manner and in a fashion capable of being appreciated by every member of a large audience. The method is being energetically pushed in America, but on this side some of the authorities have rather looked askance at it as containing the deadly germ of personal advertisement. A year or two ago a distinguished French physician who was announced to give a cinematograph demonstration at the Royal Society of Medicine found himself in an awkward fix, for while lecturer, chairman, and audience were duly assembled, it was found at the last moment that the films had been left behind!

Among the current difficulties of the profession is a short supply of motor drivers. Some of the doctors have felt that, considering the nature of their responsibilities, their drivers ought to get exemption from military service, and this view has been pressed in various local Tribunals. But, generally speaking, it has met with scant consideration, and in not a few instances the doctor has been bluntly told that he must find a woman to drive his car. In some recent cases before the Marylebone Tribunal some of the doctors demurred to this prescription. One affirmed that the women drive in a dangerous manner, and that it is too " nerve-wracking " to sit behind them. Another stated that he had made the experiment suggested to him, and with very ill success; the lady declined to live at the garage, and wished him to come for her when he contemplated a journey. But even this moving plea only secured " fourteen days' exemption."

